# Comprehensive Characterization of Integrin Subunit Genes in Human Cancers

**DOI:** 10.3389/fonc.2021.704067

**Published:** 2021-06-16

**Authors:** Kaisa Cui, Xiaohan Wu, Liang Gong, Surui Yao, Shengbai Sun, Bingxin Liu, Mingyue Zhou, Yuan Yin, Zhaohui Huang

**Affiliations:** ^1^ Wuxi Cancer Institute, Affiliated Hospital of Jiangnan University, Wuxi, China; ^2^ Laboratory of Cancer Epigenetics, Wuxi School of Medicine, Jiangnan University, Wuxi, China; ^3^ Key Laboratory of Carbohydrate Chemistry & Biotechnology, Ministry of Education, School of Biotechnology, Jiangnan University, Wuxi, China

**Keywords:** Pan-cancer, integrin subunit genes, clinical relevance, Itga11, stomach adenocarcinoma

## Abstract

Although integrin subunit genes (ITGs) have been reported to be associated with some human cancer types, a systematic assessment of ITGs across human cancers is lacking. Hence, we performed comprehensive analyses to investigate mRNA expression, copy number variation (CNV), DNA methylation, mutation, and clinical landscapes of ITGs in more than 8000 cancer patients from The Cancer Genome Atlas (TCGA) dataset. Landscapes of ITGs were established across 20 human cancer types. We observed that ITGs are extensively dysregulated with heterogeneity in different system cancer types, part of which are driven by CNV, DNA hypomethylation or mutation. Furthermore, dysregulated prognosis-related ITGs were systematically identified in each cancer type, including ITGA11 in stomach adenocarcinoma (STAD). The models based on dysregulated ITGs with clinical relevance and TNM staging indexes are good indicators in STAD and head and neck squamous cell carcinoma. Finally, ITGA11 is overexpressed and associated with poor survival in STAD cases from the TCGA and additionally Gene Expression Omnibus cohorts. Functionally, ITGA11 knockdown inhibits malignant phenotypes in STAD cell lines AGS and MKN45, demonstrating the oncogenic role of ITGA11 in STAD. Together, this study highlights the important roles of ITGs in tumorigenesis as potential prognostic biomarkers, and provide an effective resource that identifies cancer-related genes of ITGs in human cancers.

## Introduction

Cancer risk is considered a leading cause of death and an important challenge to increase life expectancy in the global world, with 19.3 million new cases and 10 million cancer deaths in 2020 (1). In the last decade, Pan-cancer analysis was used in cancer studies to obtain a comprehensive and in-depth characterization of specific functions, genes or biological processes, etc. across human cancer types (2). For instance, recently Pan-cancer analysis characterized the dual functions of heat shock proteins, which aid the development of heat shock protein inhibitors in human cancers (3). We previously established a landscape of rRNA metabolism-related genes across human cancers and explored their roles in tumorigenesis (4). Besides, another Pan-cancer analysis explored the role of ATG5 in tumor metabolism and tumor immunity (5). Hence, Pan-cancer analysis demonstrates new and unique insights in cancer researches.

Integrins, a unique class of signaling molecules, implicated in cancer progression *via* other signaling molecules, mechanotransducers, and key components of the cell migration machinery, etc. The alterations of integrin expression patterns have been observed in malignant tumors (6). Integrin subunit genes (ITGs) contains a series of alpha (ITGA1, ITGA10, ITGA11, ITGA2, ITGA2B, ITGA3, ITGA4, ITGA5, ITGA6, ITGA7, ITGA8, ITGA9, ITGAD, ITGAE, ITGAL, ITGAM, ITGAV and ITGAX) and beta (ITGB1, ITGB1BP1, ITGB1BP2, ITGB2, ITGB3, ITGB3BP, ITGB4, ITGB5, ITGB6, ITGB7, ITGB8 and ITGBL1) genes. It was reported that some ITGs were associated with epigenetic alteration and played important roles in tumor progression or cancer therapy (7–11). Although several studies reported the roles of ITGs in single cancer type (12–14), a comprehensive characterization of ITGs across human cancers has not yet been explored.

In this scenario, Pan-cancer analysis of ITGs was performed based on The Cancer Genome Atlas (TCGA) across 20 cancer types. We constructed landscapes of expression, genomic and epigenetic alterations, as well as the clinical relevance of these genes in multiple cancer types using multi-omics data. Moreover, ITGA11 was chosen to validate its oncogenic role in stomach adenocarcinoma (STAD).

## Materials and Methods

### Data and Resources

TCGA Pan-cancer data, including gene expression, copy number variation (CNV), DNA methylation, mutation and clinical data, were downloaded from the Genomic Data Commons Data Portal, including gene expression. Primary tumor samples were used in this study. Overall survival (OS) data of the TCGA cohort was obtained from the integrated TCGA Pan-cancer clinical data resource (15). Abbreviation and patient number of each cancer type were shown in **Table S1**. GSE66229, GSE13911 and GSE26899 STAD datasets were downloaded from Gene Expression Omnibus (GEO, http://www.ncbi.nlm.nih.gov/geo, **Table S2**). Expression of ITGA11 in STAD cell lines was obtained from Cancer Cell Line Encyclopedia (CCLE, https://portals.broadinstitute.org/ccle).

### Differential Expression Analysis

Gene Set Enrichment Analysis (GSEA) was used to identify the total ITGs that were significantly enriched between tumor and normal samples (16). Dysregulated ITGs were identified as previously described (4). The Jaccard index was used to evaluate the ratio of the dysregulated ITGs that are common to two cancer types. The Pheatmap package of R software 3.5.0 was used to analyze and visualize hierarchical cluster analyses.

### CNV, DNA Methylation and Mutation Analyses

Three criteria were made to identify CNV-driven dysregulated ITGs for each cancer type: (1) more than 40% tumor samples with CNV > 0.1/< -0.1 and lower than 40% tumor samples with CNV < −0.1/> 0.1; (2) the mean values of CNV in tumor samples > |0.1| (3) Pearson correlation between expression and CNV > 0.3, false discovery rate (FDR) < 0.05 was considered significant (4). Besides, we made two criteria to discover DNA methylation/hypomethylation-driven dysregulation of ITGs: (1) difference methylation levels (β-value) of the genes between tumor and normal samples >|0.05| for each cancer type. FDR<0.05 were retained; (2) Pearson correlations between gene expression levels and methylation levels <−0.3 and FDR<0.05 (4). The R software 3.5.0 was used to analyze the Pearson correlations between gene expression and CNV/methylation levels. The Maftools package from R software 3.5.0 was used to analyze mutation across human cancers from the TCGA (17). Nonsynonymous mutations were included in this study, and silent variants were excluded.

### Survival Analysis

OS analysis was performed as previously described (4, 18): the survival cases were divided into high and low expression groups for each ITGs, differences in *P* value were examined in the survival outcomes of the groups according to Kaplan–Meier survival analysis, and we select the value yielding the lowest log-rank p-values from the 10th to 90th percentiles of the samples. Hazard ratio (HR) > 1 and *P*<0.05 in the five-year were considered significantly associated with poor survival, while 0 < HR < 1 and *P* < 0.05 were regarded to be associated with favorable survival. Cox regression model analyses were conducted as previously described (18).

### Cell Lines and Transfection

STAD cell lines AGS and MKN45 were acquired from Cell Bank of Chinese Academy of Medical Sciences (Shanghai, China) and were cultured in RPMI 1640 medium containing 10% fetal bovine serum and 1% penicillin-streptomycin. All these cell lines were incubated at 37°C in 5% CO_2_. The synthetic ITGA11 siRNA#1 (5’-GACGGCAUUUGGCAUUGAATT-3’), siRNA#2 (5’-GACCUUCUCUCAGUCGAGUAUTT-3’) and the negative control siCtrl (5’-UUCUCCGAACGUGUCACGUTT-3’) (GenePharma, Shanghai, China) were transiently transfected into STAD cells using Lipofectamine 2000 (Invitrogen, USA). After 48 hours, the transfected cell lines were collected for further experiments and the efficiency of transfection was determined by quantitative RT-PCR (qRT-PCR).

### QRT-PCR

Total RNA was extracted using TRIzol reagent (TaKaRa, Japan). Complementary cDNA was generated using the Prime-Script RT reagent kit (CWBIO, China). UltraSYBR Mixture (CWBIO) was used to detect the relative mRNA expression. The levels of β-actin were used for the reference and normalized control.

### Cell Proliferation Kit 8 and Colony Formation Assays

Cells (2000) were plated in 96-well plates and grown for 5 days, detected with Cell Counting Kit-8 (CCK8, APExBIO, USA) according to the manufacturer’s instruction. Cells (2000 for AGS, 1000 for MKN45) were placed in 6-well plates and cultured in media containing 10% FBS for 10 days, and were stained with 1.0% crystal violet in 20% methanol for 30 minutes.

### Migration and Invasion Assays

Transwell assays (Corning, New York, USA) were performed to test the abilities of cell migration (1× 10^5^/well) and invasion (2 × 10^5^/well). STAD cells AGS and MKN45 were planted to the upper chamber of the 24-well insert (membrane pore size, 8 μm; Corning Life Science, USA) with or without Matrigel. The top side of the transwell chamber was filled with 10% FBS medium and the low side with 1% FBS medium. After incubation for 24 hours, cells that had migrated or invaded through the membrane were fixed with methanol for 10 minutes and stained with 0.1% crystal violet for 30 minutes.

## Results

### ITGs Were Dysregulated Across Human Cancers

**Figure 1:**
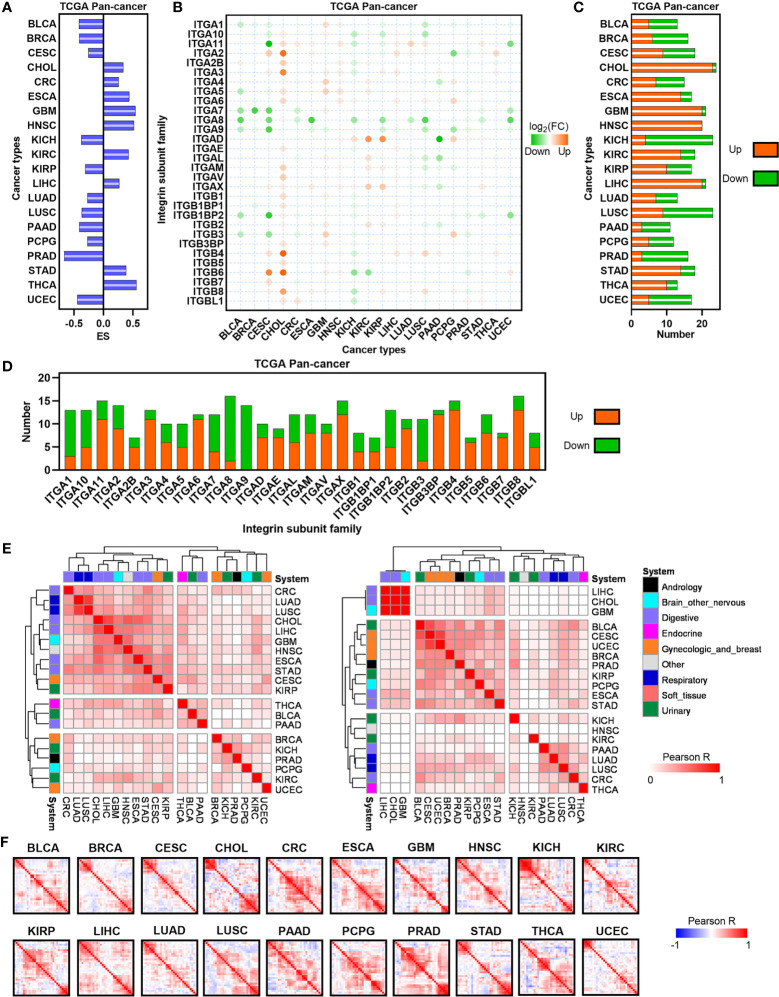
Expression landscape of ITGs in human cancers. **(A)** Barplot depicting the GSEA performance for ITGs set in TCGA tumor and normal samples.**(B)** Heatmap showing the FC of ITGs across multiple cancers from the TCGA. **(C)** The number of significantly dysregulated ITGs for each cancer from the TCGA. **(D)** Barplot showing the distribution of dysregulated ITGs across multiple cancers from the TCGA. **(E)** Heatmap visualizing the matrix of Jaccard indices of the shared connections for the upregulated (left) and downregulated (right) ITGs of each cancer from the TCGA. Hierarchical clustering was performed in the matrix. **(F)** Heatmap showing the correlation levels among ITGs in each TCGA cancer type.

To characterize the dysregulation of ITGs in human cancers, we firstly used GSEA to estimate the enrichment of total ITGs based on the TCGA dataset (16). GSEA demonstrates that total ITGs were positively enriched in CHOL, CRC, ESCA, GBM, HNSC, KIRC, LIHC, STAD and TCHA, whereas they showed negative enrichment in BLCA, BRCA, CESC, KICH, KIRP, LUAD, LUSC, PAAD, PCPG, PRAD and UCEC, suggesting that heterogenous expression ITGs across human cancers (**Figure 1A**). Next, a set of significantly dysregulated ITGs was identified in each cancer type (**Figure 1B**). Compared with other cancers, most of digestive system cancers exhibit more upregulated ITGs, such as CHOL, ESCA, LIHC and STAD (**Figure 1C**). Furthermore, we found that some ITGs such as ITGA3, ITGA6 ITGA11, ITGB4, ITGB6, etc. were frequently upregulated in human cancers, whereas ITGs including ITGA1, ITGA7, ITGA8, ITGA9 and ITGB3, etc. were usually downregulated (**Figure 1D**). To investigate the connectivity of dysregulated ITGs, we used Jaccard indexes to evaluate the overlap of these dysregulated genes in each cancer type (**Figure 1E**). For upregulated ITGs, we observed that CRC, CHOL, LIHC, ESCA, STAD, LUAD and LUSC were clustered together, suggesting the common features between digestive and respiratory system cancer types (**Figure 1E** left). For downregulated ITGs, CESC, UCEC and BRCA were clustered together, indicating gynecologic and breast cancer types with common features (**Figure 1E** right). Finally, co-expression analyses showed that ITGs were co-expressed across human cancers with different patterns (**Figure 1F** and **Supplementary Figure 1**). Our data indicate that ITGs are significantly dysregulated with heterogeneity in cancer types from different systems.

### The Genomic and Epigenetic Alterations of ITGs in Human Cancers

**Figure 2:**
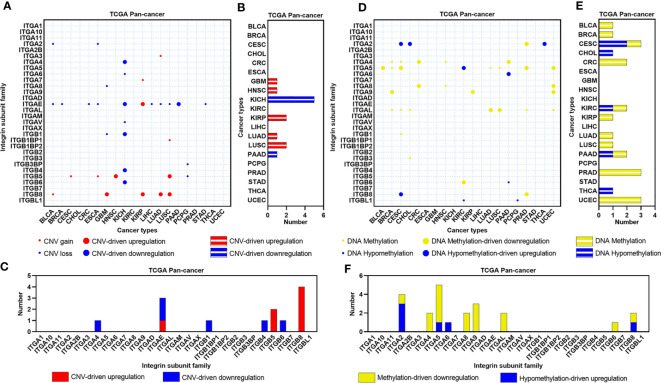
CNV and DNA methylation landscapes of ITGs in human cancers. **(A)** Heatmap showing the CNV types of ITGs across multiple cancers from the TCGA. **(B)** Barplot showing the number of CNV-driven dysregulated ITGs for each cancer from the TCGA. **(C)** Barplot showing the distribution of CNV-driven dysregulated ITGs across multiple cancers from the TCGA. **(D–F)** Similar to **(A–C)**, but for DNA methylation types and Methylation/Hypomethylation-driven dysregulated ITGs.

CNV can regulate the expression of genes related to tumorigenesis and development, we therefore evaluated the effects of CNV on the expression of ITGs in human cancers (**Figure 2A**). Seven cancer types contain CNV-driven ITGs, and KICH showed the most CNV-driven genes across human cancers (N=5, **Figure 2B**). All CNV-driven genes in KICH were CNV loss-driven, being consistent with the result of KICH that showed the most downregulated ITGs in human cancers. Eight ITGs were CNV-driven in human cancers, and ITGB8 showed a CNV-driven upregulation in four cancer types (**Figure 2C**). DNA methylation dysregulation is one of the most important epigenetic abnormalities in human cancers. Hence, we also identified the effects of DNA methylation on the expression of ITGs (**Figure 2D**). Methylation/hypomethylation-driven ITGs dysregulation were observed in more than half of cancer types analyzed in this study (**Figure 2E**), and nine ITGs appear to be DNA methylation regulated (**Figure 2F**). Interestingly, most of these ITGs demonstrate DNA methylation-driven downregulation in human cancers.

**Figure 3:**
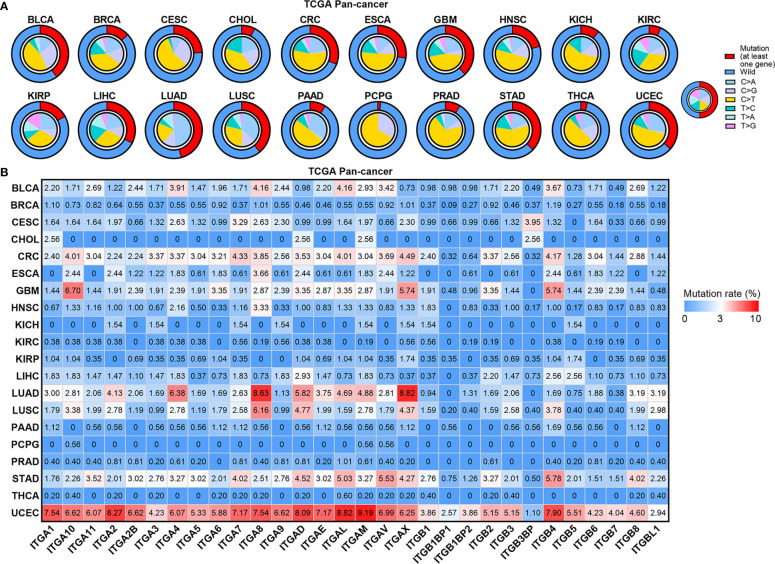
Mutation landscape of ITGs in human cancers. **(A)** Small pie charts showing the proportion of the six transition and transversion categories for each cancer type. Cycle showing the mutation rates (at least one gene mutant in this sample) for each cancer type. **(B)** Heatmap showing mutation rate of each ITG in human cancers.

Gene mutation is another important mechanism mediating carcinogenesis. Thus, we constructed the mutational landscape of ITGs in human cancers (**Figures 3A, B**). **Figure 3A** showed that CRC, ESCA, GBM, LIHC, LUAD, LUSC, STAD and UCEC had relatively higher mutational rates (more than 25%) across human cancers, whereas CHOL, KIRC, PAAD, PCPG, PRAD and THCA showed relatively lower mutation levels (less than 10%). C > T transition, a signature of base excision repair and DNA replication, was the most predominant mutation type in most cancer types. LUAD and LUSC compassed higher C > A transversions, which were supposed to be associated with tobacco carcinogens (19). Furthermore, most ITGs exhibit mutation rates less than 3% (**Figure 3B**). ITGs showed the highest mutation rates in UCEC compared with other cancer types. Note that some dysregulated genes showed relatively higher mutation rates. For instance, both ITGA8 and ITGAX were downregulated and showed more than 8% mutation rates in LUAD, whereas ITGAV and ITGB4 were upregulated and their mutation rates ranked top two in STAD. The other three ITGs, ITGA1, ITGA7 and ITGA8, were downregulated and showed more than 7% mutation rates in UCEC. We further analyzed the expression of these ITGs among normal, wild-type and mutant tumor tissues in these cancer types (**Supplementary Figure 2**). Interestingly, the expression levels of both ITGA8 and ITGAX were decreased successively in normal, wild-type and mutant LUAD samples, as well as ITGA1 and ITGA7 in UCEC (**Supplementary Figures 2A, C**). The expression of ITGB4 was increased successively in normal, wild-type and mutant LUAD samples (**Supplementary Figure 2B**). Our analyses revealed that part of the dysregulated ITGs are caused by genomic or epigenetic alteration in human cancers.

### The Expression of ITGs Is Associated With Clinical Outcomes in Human Cancers

**Figure 4:**
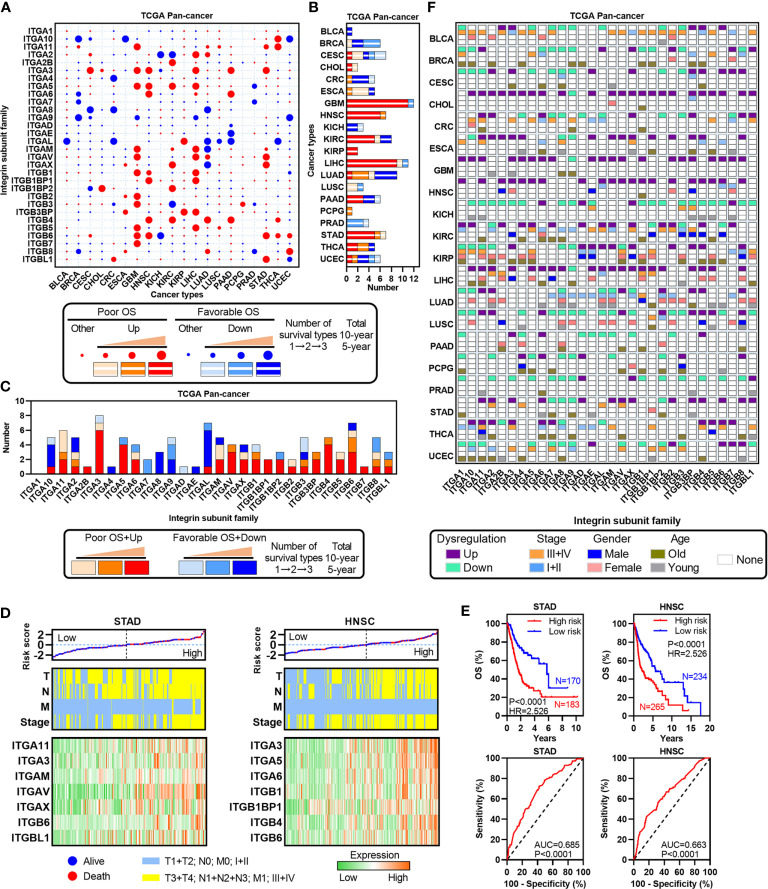
Identification of dysregulated ITGs with clinical relevance. **(A)** Heatmap showing the survival types of ITGs across multiple cancers from the TCGA. **(B)** Barplot showing the number of dysregulated ITGs with clinical relevance for each cancer from the TCGA. **(C)** Barplot showing the distribution of dysregulated ITGs with clinical relevance across multiple cancers from the TCGA. **(D)** Risk score distribution with patient OS (top, the black dotted line split the cohort into the high-risk and the low-risk score group), TNM staging indexes (middle) and expression of dysregulated ITGs with clinical relevance (bottom) in STAD and HNSC. **(E)** Kaplan-Meier plots showing the OS and ROC plots showing the AUC of the risk score in STAD and HNSC. **(F)** Heatmap showing the correlation among TNM stage, gender, age and expression of ITGs in each cancer type.

To investigate the correlation between ITGs’ expression and patients’ survival, total/10-year/5-year OSs were assessed in each cancer type (**Figure 4A**). Almost all ITGs were associated with at least one OS type. Furthermore, we identified some survival-related ITGs which were defined as dysregulated prognosis-related ITGs. We found that those upregulated and poor prognosis-related ITGs accounted for a large proportion. For instance, in GBM, HNSC, LIHC and STAD, etc., especially in HNSC or STAD, all dysregulated prognosis-related ITGs were upregulated and associated with poor OS (**Figure 4B**). ITGA11, ITGA3, ITGAV, ITGB4 and ITGB6, etc. were upregulated and associated with poor survival in multiple cancer types (**Figure 4C**). We further selected HNSC and STAD to develop Cox regression models based on the expression of dysregulated prognosis-related ITGs and TNM staging indexes, which were widely accepted as a powerful predictor for survival and treatment response in cancer therapies (**Figures 4D, E**). Our analyses showed that significant differences in prognosis was observed between the high and low risk groups in STAD or HNSC. Besides, the risk of tumor progression increased as the risk scores, and the ITGs’ expression gradually increased as the risk scores in STAD or HNSC. These data hint that the models according to the dysregulated prognosis-related ITGs and TNM staging indexes are good indicators in these two cancer types.

To further clarify the clinical significance of ITGs across human cancers, we analyzed the associations between the expression of ITGs and several clinical indexes, such as TNM stage, gender and age (**Figure 4F**). Many ITGs were associated with at least one type of three indexes. For example, ITGA11 was the poor survival factor in THCA, which is overexpressed in advanced TNM stage, male and relative younger patients. The expressions of ITGAL and ITGAM downregulated in LUAD were relative in early TNM stage, female and relative younger patients. Collectively, these results suggest that ITGs are associated with patient’s prognosis in human cancers.

### Functional Effects of ITGs in Human Cancers

**Figure 5:**
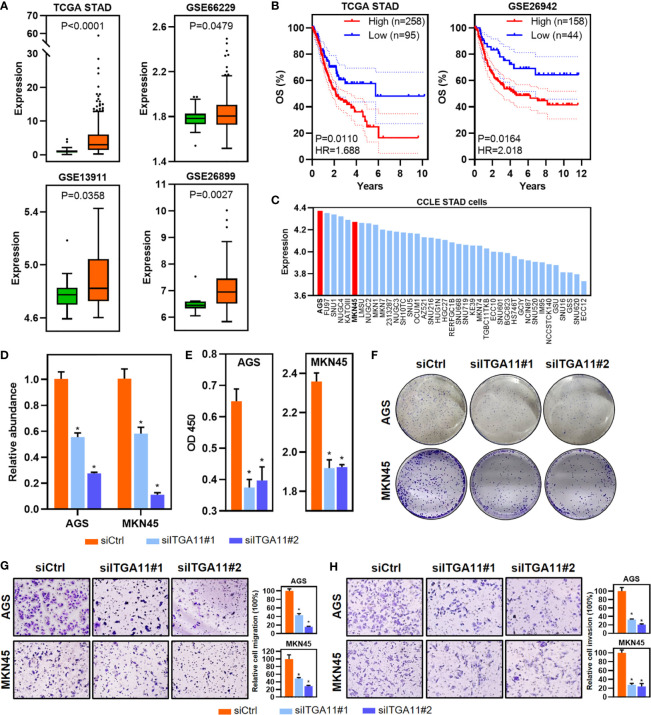
Identification and functional validation of ITGA11 in STAD. **(A)** Boxplot of ITGA11 expression in tumor and normal samples of STAD from the TCGA and GEO datasets. *P* values of boxplots are based on the Mann–Whitney test. **(B)** Kaplan–Meier plot showing 5-year OS and PFS with the expression of ITGA11 in STAD samples from the TCGA and GEO datasets. **(C)** Barplot showing the expression of ITGA11 in STAD cell lines. **(D)** Relative abundance of ITGA11 levels were detected in AGS and MKN45 by qRT-PCR after transfected siRNAs. Data are shown as means ± SEM. *P* values of barplots are based on the Mann–Whitney test. **(E, F)** The effects of ITGA11 on proliferation **(E)** and colony formation **(F)** in AGS and MKN45 cell lines. Data are shown as means ± SEM. P values of barplots are based on the Mann–Whitney test. **(G, H)** Cell migration **(G)** and invasion **(H)** were detected by transwell assays. Data are shown as means ± SEM. P values of barplots are based on the Mann–Whitney test. *P < 0.05.

STAD has a relatively higher proportion of upregulated ITGs (**Figures 1A**, **4A, B**), as well as prognosis-related ITGs. Of these dysregulated ITGs, ITGA11 was also upregulated and associated with poor prognosis in multiple cancer types, such as BRCA, GBM, LUAD, LUSC, STAD and THCA (**Figures 4A–C**). The tumor-promoting effects of ITGA11 have been reported in BRCA and non-small cell lung cancer (20, 21). However, ITGA11 has not been reported in STAD yet. We therefore selected ITGA11 for further analyses in STAD. Notably, ITGA11 was upregulated in STAD based on TCGA and additional GEO cohort (**Figure 5A**). Importantly, ITGA11 overexpression was significantly correlated with poor OS in two STAD cohorts (**Figure 5B**). Importantly, the expression of ITGA11 remained an independent prognostic variable for OS in TCGA STAD cohort (**Supplementary Figure 3A** **)**. Moreover, the expression of ITGA11 was relatively higher in advanced STAD cases from the TCGA (**Supplementary Figure 3B** **)**. To further address whether ITGA11 was associated with STAD tumorigenesis and development, we performed knockdown experiments for ITGA11 in AGS and MKN45 cell lines, in which ITGA11 was relatively overexpressed based on the data of CCLE STAD cell lines (**Figure 5C**). CCK8 assay results showed that ITGA11 knockdown significantly inhibited cell proliferation and colony formation (**Figures 5D–F**). Transwell assays showed that silencing ITGA11 expression inhibited the migration and invasion of AGS and MKN45 cells (**Figures 5G, H**). Moreover, function enrichment analysis also revealed that the pathways in cancer, epithelial-mesenchymal transition and inflammatory response signatures were enriched in ITGA11 overexpressed STADs from the two datasets (**Supplementary Figure 4**). Expression of ITGA11 was negatively correlated with CDH1, whereas positively correlated with VIM or CDH2 in tumor samples of STAD from the TCGA and GSE66229 datasets (**Supplementary Figure 5** **)**. These results suggest that ITGA11 could promote malignant phenotypes of STAD and is associated with cancer-related processes. Dysregulated prognosis-related ITGs could be an effective resource to functionally identify cancer-related genes.

## Discussion

**Figure 6:**
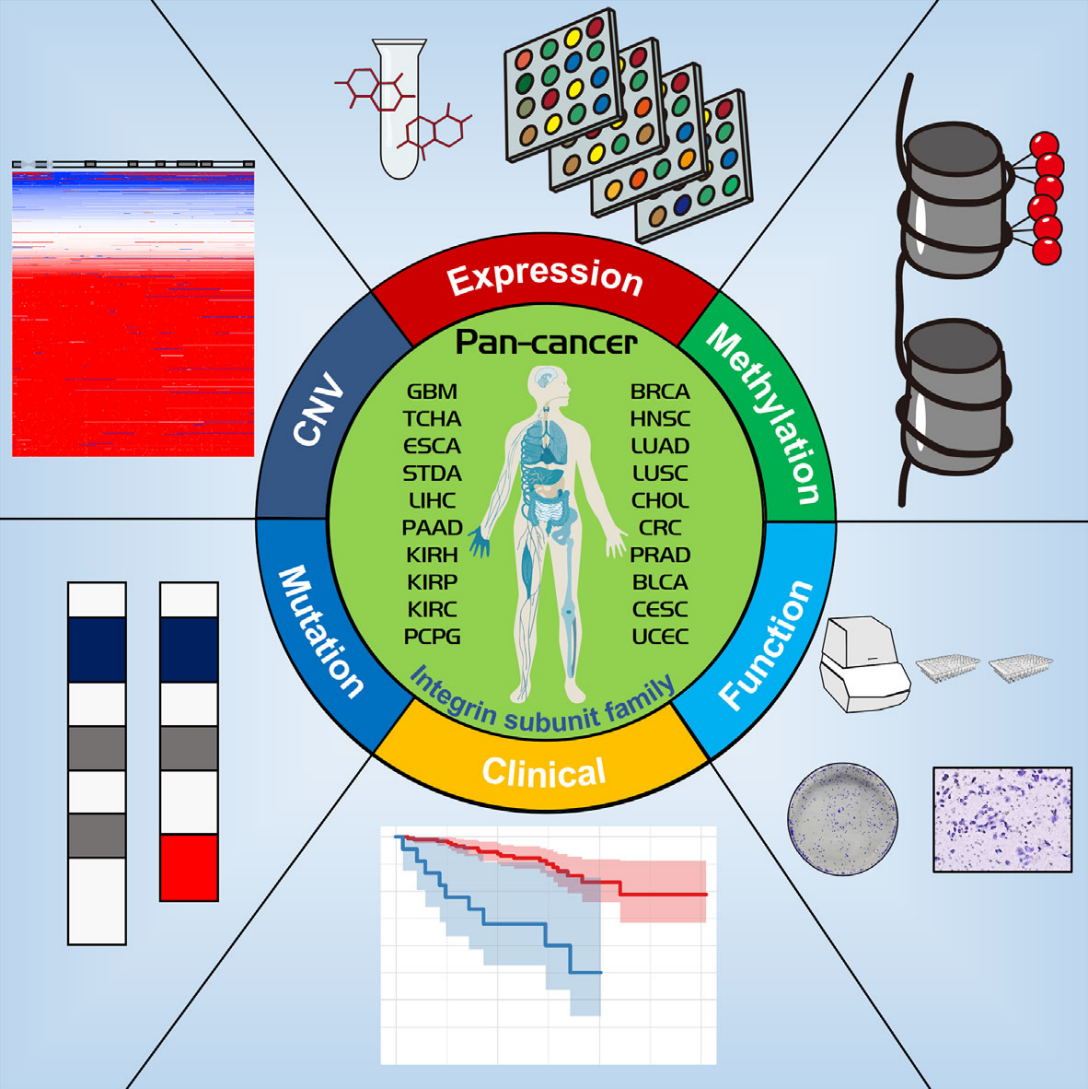
Schema demonstrates Pan-cancer analyses of ITGs in human cancers.

In this study, we performed a comprehensive analysis to explore the ITGs across 20 human cancers (**Figure 6**). We found that ITGs were usually dysregulated in human cancers. Especially different system cancer types showed different patterns. For instance, almost all the ITGs upregulated in digestive system cancer types, including CHOL, ESCA, LIHC and STAD. Interestingly, previous Pan-cancer analysis of the rRNA metabolism-related genes also exhibited similar results (4).

How are these ITGs dysregulated across human cancers? The CNV or DNA methylation levels of some genes have been reported to regulate the expression of these genes in multiple cancer types, such as FBP1 in LIHC, GABPA in BLAC (22, 23). We analyzed CNV and DNA methylation status, and revealed that part of the dysregulated ITGs were driven by CNV or DNA methylation alteration. For example, ITGA4 was identified as a DNA methylation-driven ITG in CRC and PAAD. Previous research found that DNA methylation status of ITGA4 could be severed as a biomarker in CRC (11). Besides, our mutation analysis showed that most of ITGs exhibit a less than 3% mutation rate across human cancers, except UCEC. Additionally, some ITG dysregulation was associated with their mutation, like ITGA8, ITGAX and ITGB4 in LUAD, ITGA1 and ITGA7 in UCEC. Indeed, little was known about the genomic and epigenetic alterations of ITGs, suggesting that it is urgent desired for further exploration of ITGs.

What are the consequences of the ITGs dysregulation in human cancers? Survival analysis revealed that many ITGs were associated with OS. For instance, the downregulated ITGA9 was indicated favorable OS in BRCA, CESC, GBM, LIHC and UCEC. A previous study reported that silencing ITGA9 expression inhibits liver cancer growth and metastasis *in vivo* (24). ITGB4 was upregulated and predicted poor OS in HNSC, KIRC, LUAD, PAAD and THCA. Increased expression of ITGB4 in lung cancer was correlates with poorer prognosis (10). Moreover, COX regression models indicated that some dysregulated ITGs appears to be good prognostic factors in STAD and HNSC. These results suggest that the dysregulation of ITG could predict corresponding prognosis in human cancers.

Of these identified dysregulated prognosis-related ITGS, ITGA11 was identified as a potential oncogene in BRCA, GBM, LUAD, LUSC, STAD and THCA in this study, which has been confirmed in breast and lung cancer (20, 21). However, ITGA11 has not been reported in STAD. Besides, all these ITGs with clinical relevance we identified in STAD were upregulated in tumor tissues and predicted poor survival. Hence, we selected ITGA11 for functional validation in STAD. Our results validated that ITGA11 is upregulated and associated with poor survival in STAD from additionally GEO datasets, and can promote malignant phenotypes of STAD. Furthermore, function enrichment analysis revealed that ITGA11 was associated with KEGG pathways in cancer, as well as the epithelial-mesenchymal transition process, indicating the key role of ITGA11 overexpression in tumorigenesis and progression. Besides, ITGA11 upregulation was associated with the inflammatory response process in STAD, suggesting that ITGA11 may play an important role in inflammation-mediated carcinogenesis. Inflammatory gastrointestinal diseases are associated with cancer-related mortality worldwide. Gastrointestinal cancers can be initiated by inflammation (25). Actually, previous studies demonstrated that some members of integrin β were associated with microflora and inflammatory factors in the gastric microenvironment (26, 27). These results suggest that ITGs may play important roles in inflammation-mediated carcinogenesis.

In summary, this Pan-cancer analysis constructs the landscape of ITGs in 20 cancer types. Our comprehensive characterization of ITGs may provide a foundation to expand our knowledge and further understand the role of these ITGs in human tumorigenesis and progression in the future.

## Data Availability Statement

The original contributions presented in the study are included in the article/**Supplementary Material**. Further inquiries can be directed to the corresponding author.

## Author Contributions

KC, XW, and ZH designed the study. KC, XW, and BL performed bioinformatics analyses, proofread, and visualization. KC, XW, LG, SY, SS, and MZ performed wet-lab experiments. YY provided conceptual advice. All authors discussed the results. KC, XW, LG, and ZH wrote the manuscript with comments from all authors. All authors contributed to the article and approved the submitted version.

## Funding

This work was supported by grants from the National Natural Science Foundation of China (82002550, 81672328 and 81972220), Jiangsu Key Research and Development Plan (BE2019632), Medical Key Professionals Program of Jiangsu Province (AF052141), National First-class Discipline Program of Food Science and Technology (JUFSTR20180101), Leading Talents in Medical and Health Profession of Wuxi Taihu Lake Talent Plan, and Wuxi Medical Innovation Team (CXTP003).

## Conflict of Interest

The authors declare that the research was conducted in the absence of any commercial or financial relationships that could be construed as a potential conflict of interest.

## References

[1] Sung H Ferlay J Siegel RL Laversanne M Soerjomataram I Jemal A. Global Cancer Statistics 2020: GLOBOCAN Estimates of Incidence and Mortality Worldwide for 36 Cancers in 185 Countries. CA Cancer J Clin (2021) 71:209–49. 10.3322/caac.21660 33538338

[2] Cancer Genome Atlas Research N Weinstein JN Collisson EA Mills GB Shaw KR Ozenberger BA. The Cancer Genome Atlas Pan-Cancer Analysis Project. Nat Genet (2013) 45:1113–20. 10.1038/ng.2764 PMC3919969 24071849

[3] Zhang Z Jing J Ye Y Chen Z Jing Y Li S. Characterization of the Dual Functional Effects of Heat Shock Proteins (HSPs) in Cancer Hallmarks to Aid Development of HSP Inhibitors. Genome Med (2020) 12:101. 10.1186/s13073-020-00795-6 33225964 PMC7682077

[4] Cui K Liu C Li X Zhang Q Li Y. Comprehensive Characterization of the rRNA Metabolism-Related Genes in Human Cancer. Oncogene (2020) 39:786–800. 10.1038/s41388-019-1026-9 31548613

[5] Xu C Zang Y Zhao Y Cui W Zhang H Zhu Y. Comprehensive Pan-Cancer Analysis Confirmed That ATG5 Promoted the Maintenance of Tumor Metabolism and the Occurrence of Tumor Immune Escape. Front Oncol (2021) 11:652211. 10.3389/fonc.2021.652211 33842365 PMC8027486

[6] Hamidi H Ivaska J. Every Step of the Way: Integrins in Cancer Progression and Metastasis. Nat Rev Cancer (2018) 18:533–48. 10.1038/s41568-018-0038-z PMC6629548 30002479

[7] Zhang X Song YF Lu HN Wang DP Zhang XS Huang SL. Combined Detection of Plasma GATA5 and SFRP2 Methylation Is a Valid Noninvasive Biomarker for Colorectal Cancer and Adenomas. World J Gastroenterol (2015) 21:2629–37. 10.3748/wjg.v21.i9.2629 PMC4351212 25759530

[8] Koshizuka K Hanazawa T Kikkawa N Arai T Okato A Kurozumi A. Regulation of ITGA3 by the Anti-Tumor miR-199 Family Inhibits Cancer Cell Migration and Invasion in Head and Neck Cancer. Cancer Sci (2017) 108:1681–92. 10.1111/cas.13298 PMC5543473 28612520

[9] Chen J Ji T Wu D Jiang S Zhao J Lin H. Human Mesenchymal Stem Cells Promote Tumor Growth Via MAPK Pathway and Metastasis by Epithelial Mesenchymal Transition and Integrin Alpha5 in Hepatocellular Carcinoma. Cell Death Dis (2019) 10:425. 10.1038/s41419-019-1622-1 31142737 PMC6541606

[10] Mohanty A Nam A Pozhitkov A Yang L Srivastava S Nathan A. A Non-Genetic Mechanism Involving the Integrin Beta4/Paxillin Axis Contributes to Chemoresistance in Lung Cancer. iScience (2020) 23:101496. 10.1016/j.isci.2020.101496 32947124 PMC7502350

[11] Zhang X Wan S Yu Y Ruan W Wang H Xu L. Identifying Potential DNA Methylation Markers in Early-Stage Colorectal Cancer. Genomics (2020) 112:3365–73. 10.1016/j.ygeno.2020.06.007 32531444

[12] Wu P Wang Y Wu Y Jia Z Song Y Liang N. Expression and Prognostic Analyses of ITGA11, ITGB4 and ITGB8 in Human Non-Small Cell Lung Cancer. PeerJ (2019) 7:e8299. 10.7717/peerj.8299 31875161 PMC6927340

[13] Feng C Jin X Han Y Guo R Zou J Li Y. Expression and Prognostic Analyses of ITGA3, ITGA5, and ITGA6 in Head and Neck Squamous Cell Carcinoma. Med Sci Monit (2020) 26:e926800. 10.12659/MSM.926800 33099569 PMC7594586

[14] Zhu T Chen R Wang J Yue H Lu X Li J. The Prognostic Value of ITGA and ITGB Superfamily Members in Patients With High Grade Serous Ovarian Cancer. Cancer Cell Int (2020) 20:257. 10.1186/s12935-020-01344-2 32565741 PMC7301525

[15] Liu J Lichtenberg T Hoadley KA Poisson LM Lazar AJ Cherniack AD. An Integrated TCGA Pan-Cancer Clinical Data Resource to Drive High-Quality Survival Outcome Analytics. Cell (2018) 173:400–16.e11. 10.1016/j.cell.2018.02.052 29625055 PMC6066282

[16] Subramanian A Tamayo P Mootha VK Mukherjee S Ebert BL Gillette MA. Gene Set Enrichment Analysis: A Knowledge-Based Approach for Interpreting Genome-Wide Expression Profiles. Proc Natl Acad Sci USA (2005) 102:15545–50. 10.1073/pnas.0506580102 PMC1239896 16199517

[17] Mayakonda A Lin DC Assenov Y Plass C Koeffler HP. Maftools: Efficient and Comprehensive Analysis of Somatic Variants in Cancer. Genome Res (2018) 28:1747–56. 10.1101/gr.239244.118 PMC6211645 30341162

[18] Cui K Yao S Zhang H Zhou M Liu B Cao Y. Identification of an Immune Overdrive High-Risk Subpopulation With Aberrant Expression of FOXP3 and CTLA4 in Colorectal Cancer. Oncogene (2021) 40:2130–45. 10.1038/s41388-021-01677-w 33627780

[19] Alexandrov LB Nik-Zainal S Wedge DC Aparicio SA Behjati S Biankin AV. Signatures of Mutational Processes in Human Cancer. Nature (2013) 500:415–21. 10.1038/nature12477 PMC3776390 23945592

[20] Primac I Maquoi E Blacher S Heljasvaara R Van Deun J Smeland HY. Stromal Integrin alpha11 Regulates PDGFR-Beta Signaling and Promotes Breast Cancer Progression. J Clin Invest (2019) 129:4609–28. 10.1172/JCI125890 PMC6819106 31287804

[21] Ando T Kage H Matsumoto Y Zokumasu K Yotsumoto T Maemura K. Integrin Alpha11 in Non-Small Cell Lung Cancer Is Associated With Tumor Progression and Postoperative Recurrence. Cancer Sci (2020) 111:200–8. 10.1111/cas.14257 PMC6942423 31778288

[22] Hirata H Sugimachi K Komatsu H Ueda M Masuda T Uchi R. Decreased Expression of Fructose-1,6-bisphosphatase Associates With Glucose Metabolism and Tumor Progression in Hepatocellular Carcinoma. Cancer Res (2016) 76:3265–76. 10.1158/0008-5472.CAN-15-2601 27197151

[23] Guo Y Yuan X Li K Dai M Zhang L Wu Y. GABPA Is a Master Regulator of Luminal Identity and Restrains Aggressive Diseases in Bladder Cancer. Cell Death Differ (2020) 27:1862–77. 10.1038/s41418-019-0466-7 PMC7244562 31802036

[24] Zhang YL Xing X Cai LB Zhu L Yang XM Wang YH. Integrin Alpha9 Suppresses Hepatocellular Carcinoma Metastasis by Rho GTPase Signaling. J Immunol Res (2018) 2018:4602570. 10.1155/2018/4602570 29951557 PMC5989280

[25] Sukocheva OA Furuya H Ng ML Friedemann M Menschikowski M Tarasov VV. Sphingosine Kinase and Sphingosine-1-Phosphate Receptor Signaling Pathway in Inflammatory Gastrointestinal Disease and Cancers: A Novel Therapeutic Target. Pharmacol Ther (2020) 207:107464. 10.1016/j.pharmthera.2019.107464 31863815

[26] Pick R Begandt D Stocker TJ Salvermoser M Thome S Böttcher RT. Coronin 1A, A Novel Player in Integrin Biology, Controls Neutrophil Trafficking in Innate Immunity. Blood (2017) 130(7):847–58. 10.1182/blood-2016-11-749622 28615221

[27] Feige MH Sokolova O Pickenhahn A Maubach G Naumann M. HopQ Impacts the Integrin α5β1-Independent NF-κB Activation by Helicobacter Pylori in CEACAM Expressing Cells. Int J Med Microbiol (2018) 308(5):527–33. 10.1016/j.ijmm.2018.05.003 29779861

