# Supplementary material for: Comprehensive Characterization of Integrin Subunit Genes in Human Cancers
Source: Front Oncol. 2021 Jun 16;11:704067. doi: 10.3389/fonc.2021.704067 (PMC8242346; doi:10.3389/fonc.2021.704067)
Supplement: Supplementary file 1 [file Image_1.pdf]

Comprehensive characterization of integrin subunit genes in human cancers

Supplementary Figures

Figure. S1 Related to Figure. 1F 2
Figure. S2 Related to Figure. 3 3
Figure. S3 Related to Figure. 5 4
Figure. S4 Related to Figure. 5 5
Figure. S5 Related to Figure. 5 6

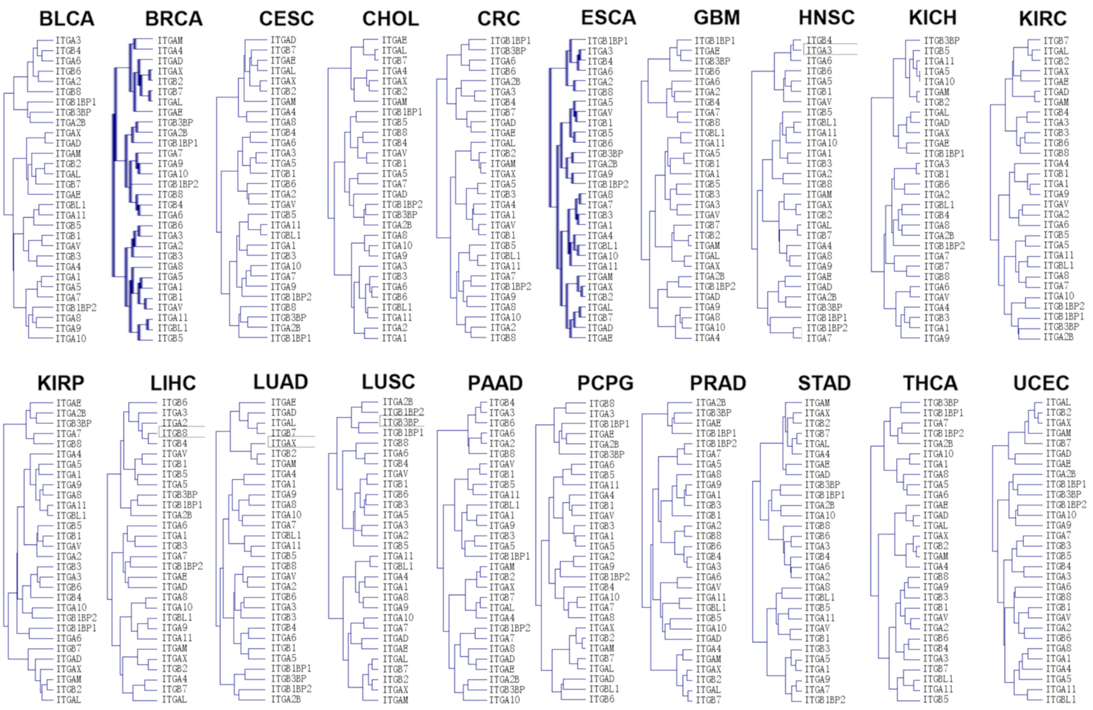

Figure. S1 Related to Figure. 1F

The gene list of gene trees in Figure.1F.

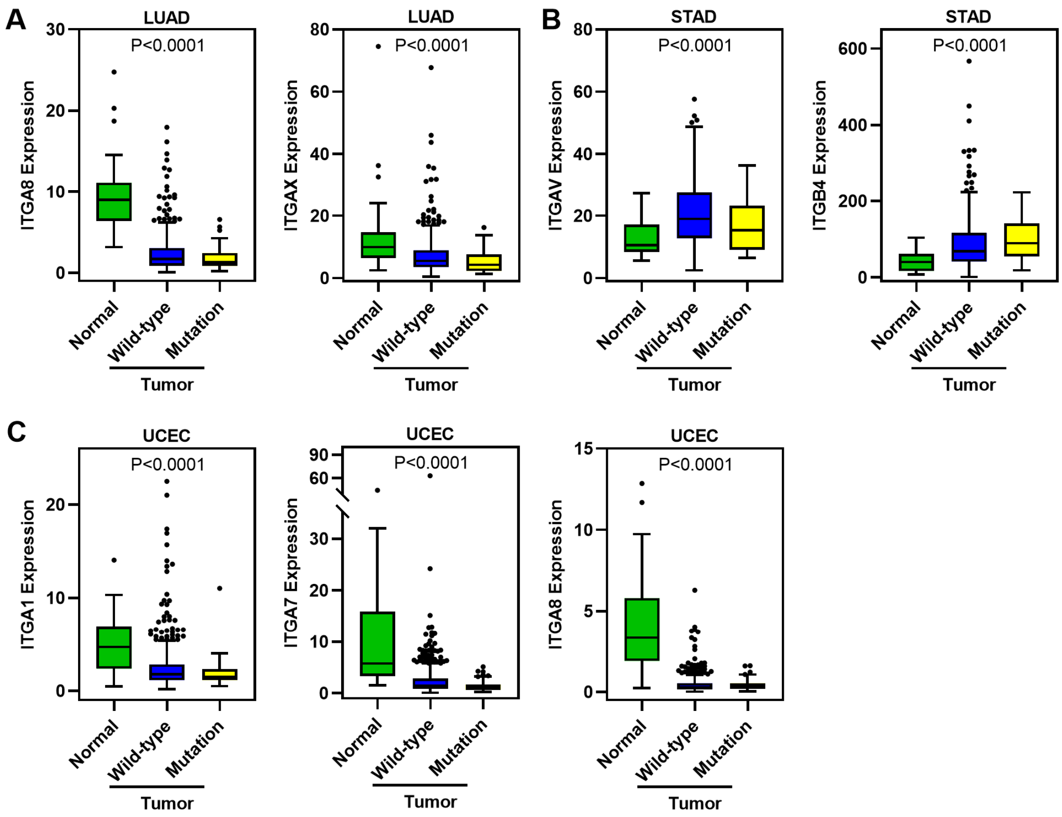

Figure. S2 Related to Figure. 3

(A) Boxplot showing the expression of ITGA8 and ITGAX group by normal, wild-type and mutant samples in LUAD. **(B)** Boxplot showing the expression of ITGAV and ITGB4 for normal, wild-type and mutant samples in STAD. **(C)** Boxplot showing the expression of ITGA1, ITGA7 and ITGA8 grouped by normal, wild-type and mutant samples in UCEC. Boxplots show median, quartiles, min and max. *P* values of boxplots are based on the Kruskal–Wallis test.

A

Univariable Cox				
		P	HR	95% CI
ITGA11	High vs Low	**0.012**	**1.683**	**1.121-2.525**
T	T4+T3 vs T2+T1	0.040	1.517	1.020-2.257
N	N3+N2+N1 vs N0	0.008	1.709	1.153-2.533
M	M1 vs M0	0.014	2.056	1.160-3.642
Stage	IV+III vsII+I	0.003	1.669	1.190-2.340
Multivariable Cox				
		P	HR	95% CI
ITGA11	High vs Low	**0.01**	**1.713**	**1.137-2.581**
T	T4+T3 vs T2+T1	0.637	1.116	0.708-1.757
N	N3+N2+N1 vs N0	0.169	1.426	0.860-2.365
M	M1 vs M0	0.074	1.715	0.949-3.098
Stage	IV+III vsII+I	0.43	1.214	0.750-1.966

B

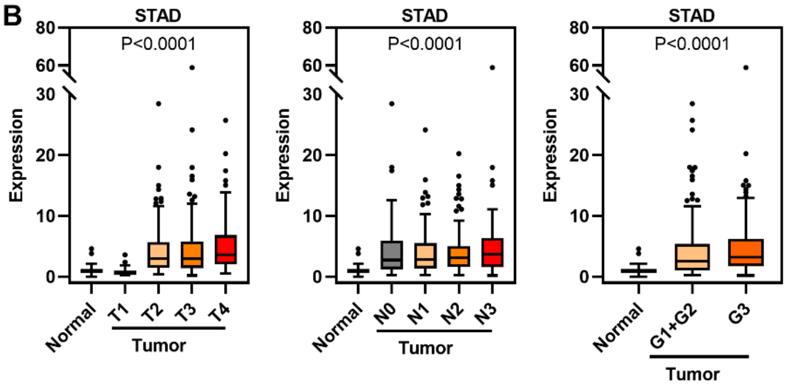

Figure. S3 Related to Figure. 5

(A) Cox survival models for ITGA11 and TNM staging indexes in TCGA STAD dataset. **(B)** Boxplot showing the expression of ITGA11 grouped by T stage, N stage and tumor grade in STAD samples from the TCGA dataset. Boxplots show median, quartiles, min and max. *P* values of boxplots are based on the Kruskal–Wallis test.

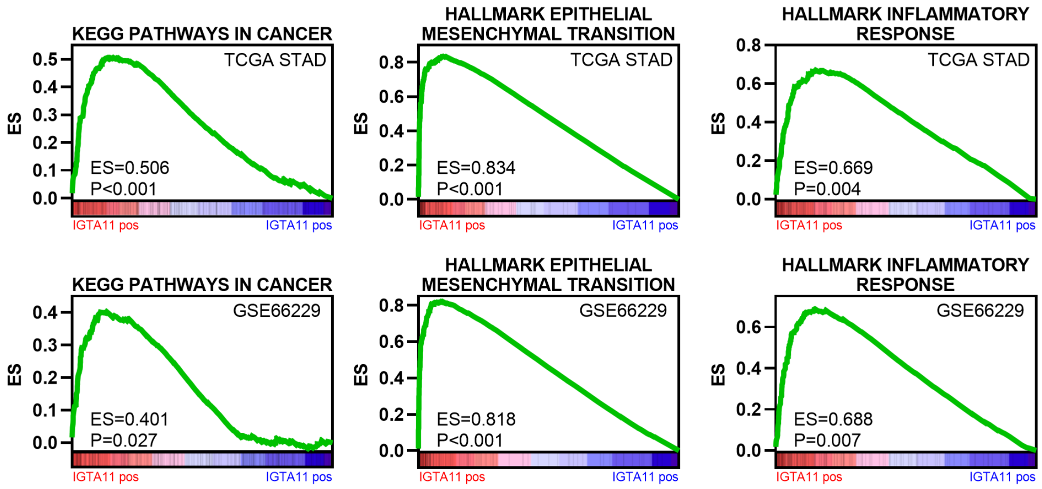

Figure. S4 Related to Figure. 5

GSEA plot for of KEGG PATHWAYS IN CANCER, HALLMARK EPITHELIAL MESENCHYMAL TRANSITION and HALLMARK INFLAMMATORY RESPONSE signatures in tumor samples of STAD from the TCGA and GSE66229 datasets based on ITGA11 expression.

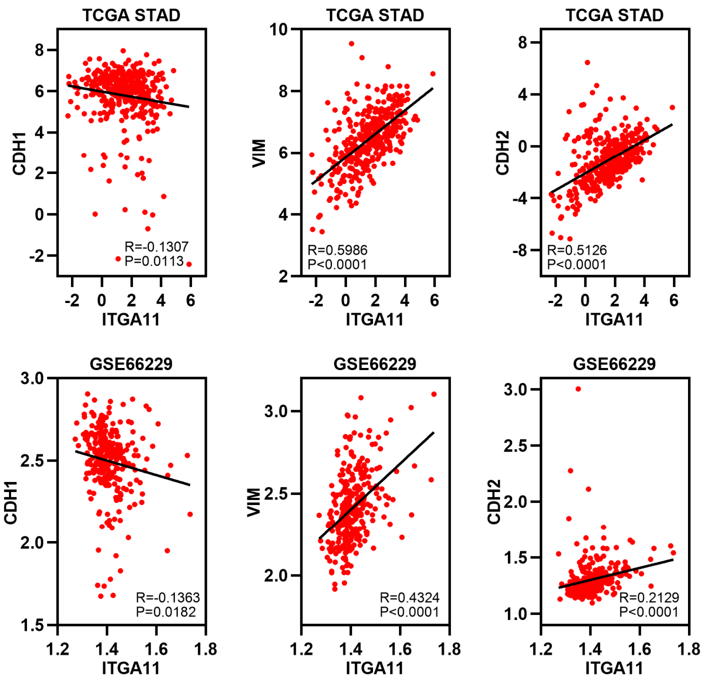

Figure. S5 Related to Figure. 5

Scatterplot for the correlation between expression of ITGA11 and representative EMT markers in tumor samples of STAD from the TCGA and GSE66229 datasets.
